# A Population-Based Evaluation of a Publicly Funded, School-Based HPV Vaccine Program in British Columbia, Canada: Parental Factors Associated with HPV Vaccine Receipt

**DOI:** 10.1371/journal.pmed.1000270

**Published:** 2010-05-04

**Authors:** Gina Ogilvie, Maureen Anderson, Fawziah Marra, Shelly McNeil, Karen Pielak, Meena Dawar, Marilyn McIvor, Thomas Ehlen, Simon Dobson, Deborah Money, David M. Patrick, Monika Naus

**Affiliations:** 1 University of British Columbia, Vancouver, Canada; 2 BC Centre for Disease Control, Vancouver, Canada; 3 Dalhousie University, Halifax, Canada; National Institute of Child Health and Human Development, United States of America

## Abstract

**Background:**

Information on factors that influence parental decisions for actual human papillomavirus (HPV) vaccine receipt in publicly funded, school-based HPV vaccine programs for girls is limited. We report on the level of uptake of the first dose of the HPV vaccine, and determine parental factors associated with receipt of the HPV vaccine, in a publicly funded school-based HPV vaccine program in British Columbia, Canada.

**Methods and Findings:**

All parents of girls enrolled in grade 6 during the academic year of September 2008–June 2009 in the province of British Columbia were eligible to participate. Eligible households identified through the provincial public health information system were randomly selected and those who consented completed a validated survey exploring factors associated with HPV vaccine uptake. Bivariate and multivariate analyses were conducted to calculate adjusted odds ratios to identify the factors that were associated with parents' decision to vaccinate their daughter(s) against HPV. 2,025 parents agreed to complete the survey, and 65.1% (95% confidence interval [CI] 63.1–67.1) of parents in the survey reported that their daughters received the first dose of the HPV vaccine. In the same school-based vaccine program, 88.4% (95% CI 87.1–89.7) consented to the hepatitis B vaccine, and 86.5% (95% CI 85.1–87.9) consented to the meningococcal C vaccine. The main reasons for having a daughter receive the HPV vaccine were the effectiveness of the vaccine (47.9%), advice from a physician (8.7%), and concerns about daughter's health (8.4%). The main reasons for not having a daughter receive the HPV vaccine were concerns about HPV vaccine safety (29.2%), preference to wait until the daughter is older (15.6%), and not enough information to make an informed decision (12.6%). In multivariate analysis, overall attitudes to vaccines, the impact of the HPV vaccine on sexual practices, and childhood vaccine history were predictive of parents having a daughter receive the HPV vaccine in a publicly funded school-based HPV vaccine program. By contrast, having a family with two parents, having three or more children, and having more education was associated with a decreased likelihood of having a daughter receive the HPV vaccine.

**Conclusions:**

This study is, to our knowledge, one of the first population-based assessments of factors associated with HPV vaccine uptake in a publicly funded school-based program worldwide. Policy makers need to consider that even with the removal of financial and health care barriers, parents, who are key decision makers in the uptake of this vaccine, are still hesitant to have their daughters receive the HPV vaccine, and strategies to ensure optimal HPV vaccine uptake need to be employed.

*Please see later in the article for the Editors' Summary*

## Abstract 2

Analysis of a telephone survey by Gina Ogilvie and colleagues identifies the parental factors associated with HPV vaccine uptake in a school-based program in Canada.

## Editors' Summary

**Background:**

About 10% of cancers in women occur in the cervix, the structure that connects the womb to the vagina. Every year, globally, more than a quarter of a million women die because of cervical cancer, which only occurs after the cervix has been infected with a human papillomavirus (HPV) through sexual intercourse. There are many types of HPV, a virus that infects the skin and the mucosa (the moist membranes that line various parts of the body, including the cervix). Although most people become infected with HPV at some time in their life, most never know they are infected. However, some HPV types cause harmless warts on the skin or around the genital area and several—in particular, HPV 16 and HPV 18, so-called high-risk HPVs—can cause cervical cancer. HPV infections are usually cleared by the immune system, but about 10% of women infected with a high-risk HPV develop a long-term infection that puts them at risk of developing cervical cancer.

**Why Was This Study Done?:**

Screening programs have greatly reduced cervical cancer deaths in developed countries in recent decades by detecting the cancer early when it can be treated; but it would be better to prevent cervical cancer ever developing. Because HPV is necessary for the development of cervical cancer, vaccination of girls against HPV infection before the onset of sexual activity might be one way to do this. Scientists recently developed a vaccine that prevents infection with HPV 16 and HPV 18 (and with two HPVs that cause genital warts) and that should, therefore, reduce the incidence of cervical cancer. Publicly funded HPV vaccination programs are now planned or underway in several countries; but before girls can receive the HPV vaccine, parental consent is usually needed, so it is important to know what influences parental decisions about HPV vaccination. In this study, the researchers undertake a telephone survey to determine the uptake of the HPV vaccine by 11-year-old girls (grade 6) in British Columbia, Canada, and to determine the parental factors associated with vaccine uptake; British Columbia started a voluntary school-based HPV vaccine program in September 2008.

**What Did the Researchers Do and Find?:**

In early 2009, the researchers contacted randomly selected parents of girls enrolled in grade 6 during the 2008–2009 academic year and asked them to complete a telephone survey that explored factors associated with HPV vaccine uptake. 65.1% of the 2,025 parents who completed the survey had consented to their daughter receiving the first dose of HPV vaccine. By contrast, more than 85% of the parents had consented to hepatitis B and meningitis C vaccination of their daughters. Nearly half of the parents surveyed said their main reason for consenting to HPV vaccination was the effectiveness of the vaccine. Conversely, nearly a third of the parents said concern about the vaccine's safety was their main reason for not consenting to vaccination and one in eight said they had been given insufficient information to make an informed decision. In a statistical analysis of the survey data, the researchers found that a positive parental attitude towards vaccination, a parental belief that HPV vaccination had limited impact on sexual practices, and completed childhood vaccination increased the likelihood of a daughter receiving the HPV vaccine. Having a family with two parents or three or more children and having well-educated parents decreased the likelihood of a daughter receiving the vaccine.

**What Do These Findings Mean?:**

These findings provide one of the first population-based assessments of the factors that affect HPV vaccine uptake in a setting where there are no financial or health care barriers to vaccination. By identifying the factors associated with parental reluctance to agree to HPV vaccination for their daughters, these findings should help public-health officials design strategies to ensure optimal HPV vaccine uptake, although further studies are needed to discover why, for example, parents with more education are less likely to agree to vaccination than parents with less education. Importantly, the findings of this study, which are likely to be generalizable to other high-income countries, indicate that there is a continued need to ensure that the public receives credible, clear information about both the benefits and long-term safety of HPV vaccination.

**Additional Information:**

Please access these Web sites via the online version of this summary at http://dx.doi.org/10.1371/journal.pmed.1000270.

- The US National Cancer Institute provides information about cervical cancer for patients and for health professionals, including information on HPV vaccines (in English and Spanish)
- The US Centers for Disease Control and Prevention also has information about cervical cancer and about HPV
- The UK National Health Service Choices website has pages on cervical cancer and on HPV vaccination
- More information about cervical cancer and HPV vaccination is available from the Macmillan cancer charity
- ImmunizeBC provides general information about vaccination and information about HPV vaccination in British Columbia
- MedlinePlus provides links to additional resources about cervical cancer (in English and Spanish)

## Introduction

The vaccine for the human papillomavirus (HPV) is an important tool in the prevention of cervical cancer [1]–[5]. In order to maximize the benefit of the HPV vaccine for cervical cancer prevention and for programs to be cost-effective, vaccine programs should be offered to girls prior to the commencement of sexual activity [6]–[8]. Because of the age at which the HPV vaccine is given in many jurisdictions, parents will often need to provide consent. Careful reflection on parents' perspectives and concerns about this vaccine is essential in order to ensure optimal uptake rates. Studies on parental attitudes and intention-to-vaccinate have shown that despite the outstanding clinical efficacy and reassuring side-effect profile of this vaccine, concerns remain about the vaccine and about the willingness of parents to have their daughters receive HPV vaccination [9]–[18]. In a recent systematic review on the topic, global HPV vaccine acceptability among parents ranged from 54.9% to 81.0% [19], and studies have highlighted issues such as vaccine safety, impact on sexual practices, age of daughter, awareness of HPV, education, and cervical cancer screening history among many others as key predictors of HPV vaccine acceptance. However, most studies have primarily focused on factors predicting parental intention to have a daughter receive the HPV vaccines and were conducted prior to the approval of the HPV vaccine or implementation of a publicly funded vaccine program. In contrast, data on factors influencing parental decisions for actual or real HPV vaccine receipt in publicly funded and delivered vaccine programs for girls is limited [20]. As publicly funded HPV vaccines programs are now being planned it is critical that parental factors associated with actual uptake of the HPV vaccine are understood.

In Canada, health falls under provincial/territorial jurisdiction and by September 2009, all of the 14 provinces and territories in Canada commenced a school-based HPV vaccine program. In September 2008, the province of British Columbia in Canada embarked on a voluntary, school-based HPV vaccination program for girls in grade 6 (aged 11 y) and grade 9 (aged 14 y) with Gardasil. With the implementation of this program, and given the critical role of parents in vaccine uptake and previous research that indicated that British Columbians were less likely than Atlantic Canadians to intend to have their daughters receive the HPV vaccine [14], we took the opportunity to conduct a population-based evaluation of the HPV vaccine program in the province. We conducted a telephone survey of a random selection of parents of grade 6 girls in the province who were eligible to receive the HPV vaccine. The objective of this evaluation was to assess the level of uptake of the first dose of the HPV vaccine and to determine the factors associated with receipt of the HPV vaccine.

## Methods

### Participants and Data Collection

All parents of girls enrolled in grade 6 during the academic year of September 2008–June 2009 in the province of British Columbia were eligible to participate. Telephone numbers of eligible households were identified through the integrated Public Health information system (iPHIS) program. iPHIS is a software and public health information system used by 14 of 16 Health Service Delivery Areas of British Columbia for notifiable disease reporting, as an immunization registry, and for vaccine-associated adverse event reporting. iPHIS contains identifiers of all individuals who have received a public health service, including well baby examination, hearing and vision screening, and immunization services. Phone numbers of households with a girl in grade 6 in the province were identified as part of a comprehensive HPV vaccine program evaluation, and households were randomly selected to be contacted by telephone after the first dose of the HPV vaccine had been offered through the school-based program and invited to participate in this survey. Parents who consented were interviewed by trained, experienced research staff. The evaluation received ethical approval from University of British Columbia and funding from the BC Centre for Disease Control.

### HPV Vaccine Program in British Columbia

In British Columbia, all vaccines provided in schools, including the HPV vaccine, are fully funded by the public health program in the province. The vaccines are delivered as part of a comprehensive school-based vaccination program for hepatitis B, meningococcal C, tetanus-diphtheria, and acellular pertussis booster, as well as a catch-up program for varicella zoster virus vaccine. In 2008, Gardasil was added to the school-based vaccine program in British Columbia. Trained public health nurses offer these vaccines in all public and independent schools through the entire province free of cost, and in the grade 6 program, parents provide consent for their daughters to receive, or not receive, HPV and other vaccines. Children who are absent are able to receive vaccines on days when the school nurses return for other classes, or can attend local public health units to receive the vaccine free of charge. Education for the HPV vaccine program focused on cervical cancer prevention, and was widely promoted through the schools with information packages and DVDs aimed at parents and children. Public health nurses offered local educational sessions where possible. Parents were also provided with the link to www.immunizeBC.ca, which has extensive information on all vaccines, including HPV.

### Theoretical Model

The survey tool is based on the theoretical model of Theory of Planned Behaviour (TPB) [21]. This psychological model of behaviour change examines how human action is guided and distils the elements that contribute to an actual behaviour (in this case, consent to have a daughter receive the HPV vaccine), or the most proximate measure of change, behaviour intention. According to TPB, behaviours or behavioural intentions are a result of attitudes, subjective norms, and perceived behavioural control. This well-established model provides a foundation for questionnaire development regarding health behaviours or behaviour intentions. For this survey, we will examine the actual behaviour (receipt of the vaccine) and discern parental factors that predict vaccine uptake.

### Survey Instrument Development

Questionnaire development adhered to the steps needed to construct a TPB questionnaire and was based on a previous study on intention to vaccinate [14]. The “population of interest” was defined as parents of daughters in grade 6 in British Columbia, and the “behaviour under examination” was parental consent (or not) to have daughters receive the HPV vaccine. Behaviour was measured by parental self-report as to whether or not they had consented to have their daughter receive the HPV vaccine. Perceived advantages and disadvantages of the HPV vaccine, most important people/groups who would approve or disapprove of the vaccine, and perceived barriers/facilitating factors were identified through a comprehensive literature search, an elicitation survey of ten parents to determine factors influential in their decision to immunize or not to immunize their daughter(s) against HPV, and results from intention to vaccinate studies [14]. A draft survey including all constructs was pilot tested with parents to ensure comprehension and to ensure no domains of relevance had been missed. Parents identified questions on “barriers/facilitating factors” for this vaccine program that were redundant and confusing, as this was a publicly funded, provincial program delivered at every school by school nurses, thus removing any expected barriers such as cost and access to the program/practitioners.

### Survey Content

Demographics items assessed included age and gender of respondent, region of residence, age(s) and number of daughters, respondent education, cultural background, history of abnormal Pap smears or cervical cancer, religious affiliation, and family composition. Participants were asked about adherence to childhood vaccination schedules and knowledge of cervical cancer and HPV at the start of the survey. Participants were next asked to report whether or not their daughter had received the hepatitis B, meningococcal C, and HPV vaccine that year, as well as the number of doses of the HPV vaccine received, and intention to complete the series for the HPV vaccine. Parents were asked to provide the main reason for electing to have their daughter receive or not receive the HPV vaccine, as well as any reason for their choice, and these reasons were categorized. Participants were asked about specific psychological constructs that could influence their decision to vaccinate or not vaccinate their daughter with the HPV vaccine. In keeping with TPB, these constructs included attitudes toward vaccines in general and the HPV vaccine in particular, perceived impact of the HPV vaccine on their daughter's sexual practices, and the seriousness of HPV infection and cervical cancer as diseases. These constructs were assessed using seven-point Likert scales (1, strongly disagree; 4, neutral; 7, strongly agree) with four or five items per construct.

### Sampling Frame and Telephone Recruitment

British Columbia is the most western province of Canada, with a population of more than 4 million. It is divided into five geographic health authorities and each health authority is divided into health service delivery areas (HSDAs). There are a total of 16 HSDAs in the province, and each health authority has either three or four HSDAs. Two of the HSDAs, which include ∼15% of the eligible girls in the province, do not use iPHIS, the provincial immunization registry, as their public health information system and thus were not included in the sampling frame. In order to ensure a representative sample from across the province, we generated a sampling frame from British Columbia population estimates for each of the five geographic health authorities of 11-y-old girls for 2008 from Population Extrapolation for Organization Planning with Less Error, run cycle 32 (P.E.O.P.L.E. 32) [22], excluding the two HSDAs not participating. P.E.O.P.L.E. 32 is the subprovincial (local health authority, health region, regional district, and development region) population projections that are released annually by the BC government (BC Stats). P.E.O.P.L.E. 32 was released in 2007. Assuming a population of 20,000 girls in the eligible age cohort, response rate of 50%, and a 95% confidence interval (CI) of ±2%, we needed to recruit 2,144 participants [23]. We randomly selected participants from the datasets from each health authority, to ensure that at the end of the evaluation we had a representative sample based on the population size of 11-y-old girls in each health authority in the province.

Telephone calls for the evaluation were conducted by an experienced research company who had carried out previous parental attitudinal surveys in British Columbia. Participants were randomly selected from each health authority, and households were contacted in the random order provided. Households were called a maximum of four times, with attempts to contact made in the morning, afternoon, evening, and Saturdays. We stopped calling households once one of the following occurred: participant declined; number not in service; no answer after four attempts; messages left four times; or survey not completed/ineligible.

### Analysis

Descriptive analyses of sample demographics were conducted. Item reliability was established for psychological construct scales using Cronbach's alpha, and mean values for each scale were calculated. For scale items, composite scale scores were calculated and dichotomized with a mean value of 4.5 as a cut-off, with scores ≥4.5 indicating a general positive value for the HPV vaccine (i.e., a positive attitude to vaccines, belief that the HPV vaccine had limited influence on sexual behaviour). Composite variables were created for the predictor variables and dichotomized, and then entered into the model as described in the methods. Bivariate analyses were conducted using Chi-square comparing the responses of parents who vaccinated their daughter(s) against HPV to those who did not vaccinate. Variables that achieved *p*<0.05 were offered for inclusion in a multivariable model to achieve a best fit model. Logistic regression was conducted to calculate adjusted odds ratios to identify the factors that were associated with parents' decision to vaccinate their daughter(s) against HPV. Backwards logistic regression analysis was conducted to calculate adjusted odds ratios to identify the factors that were associated with parents' decision to vaccinate their daughter(s) against HPV. We also used additional backwards and forward variable selection techniques to confirm that the model and findings were robust (unpublished data). Analyses were conducted with SPSS version 14.0 for Windows.

## Results

This program evaluation was carried out between January 18, 2009, and March 19, 2009, 4 mo after the provincial HPV vaccine program commenced. Of the 23,614 girls in grade 6 in the province of British Columbia, contact information was available for 20,161 from 14 of 16 health service areas (85.4%) in iPHIS. 5,489 of 20,161 eligible households, stratified by health authority, were randomly contacted by the research team. Of the 4,335 numbers in service (78.9%), 304 did not speak English. Of the remaining 4,031 eligible to complete the survey, 2,025 parents agreed to complete the survey (50.2%).

Demographic characteristics of the participants are shown on Table 1. The majority of survey respondents were female (84.9%), most had given their daughters all childhood vaccinations (94.1%), and more than 90% had heard of HPV. Respondents were representative of the population distribution of grade 6 girls in health authorities in the province, and 1,318 (65.1%; 95% CI 63.1–67.1) of parents in the survey reported that their daughters had received the first dose of the HPV vaccine. In the same school-based vaccine program, 1,790 (88.4%; 95% CI 87.1–89.7) reported consenting to the hepatitis B vaccine, and 1,751 (86.5%; 95% CI 85.1–87.9) consented to the meningitis C vaccine. In those who received the first dose of the HPV vaccine, 97.5% said that they planned to have their daughter receive the next dose of the HPV vaccine. Of the 34.9% of parents who did not consent to have daughters receive the HPV vaccine, almost 50% stated that they would prefer to have their daughter receive the HPV vaccine in the future.

**Table 1:** Demographic characteristics of survey respondents.

Characteristics of Respondents (*n* = 2,025)	*n* (%)
**Respondents' gender**	
Female	1,719 (84.9)
Male	301 (14.9)
No response	5 (0.2)
**Age of respondents (y)**	
19–29	17 (0.8)
30–39	632 (31.2)
40–49	1,135 (56.0)
50–59	189 (9.3)
60+	15 (0.7)
No response	37 (1.8)
**Child received all childhood vaccines**	
Yes (all)	1,903 (94.1)
Yes (some)	82 (4.1)
Unsure	8 (0.4)
No	30 (1.5)
**Ever heard of HPV**	
Yes	1,878 (92.7)
No	147 (7.3)
**History of cervical cancer (self or partner)**	
Yes	80 (4.0)
No	1,906 (94.1)
Unsure/missing	39 (1.9)
**History of abnormal Pap smear (self or partner)**	
Yes	700 (34.6)
No	1,274 (62.9)
Unsure/missing	51 (2.5)
**Education**	
High school education/vocational school	713 (35.9)
Some or complete undergraduate degree	1,119 (55.3)
Postgraduate degree	156 (7.7)
Missing	37 (.8)
**Family composition**	
Single parent/guardian	252 (12.4)
Two parents (male/female)	1,513 (74.7)
Parents/guardians extended family	92 (4.5)
Blended families	128 (6.3)
Missing	40 (2.0)
**Number of children**	
One or two children	1,297 (64.0)
Three of more children	728 (36.0)
**Country of birth**	
Canada	1,544 (76.2)
England	54 (2.7)
China	15 (0.7)
India	64 (3.2)
Philippines	39 (1.9)
United States	47 (2.3)
Germany	16 (0.8)
Other	246 (12.1)
**Religious background**	
Christian (Catholic or Protestant)	327 (16.2)
Christian (other)	440 (21.7)
Sikh	47 (2.3)
Muslim	18 (0.9)
Buddhist	12 (0.6)
Evangelical Christian	8 (0.3)
Jewish	3 (0.1)
Other religion (including other Christian denominations)	476 (23.5)
None	694 (43.3)
**Organized religion**	
No religious affiliation	632 (31.2)
Religious affiliation	1,393 (68.8)

Parents were asked to list both a main (single) reason and any reason for their vaccine choice. The main reasons for having a daughter receive the HPV vaccine were the effectiveness of the vaccine (48.0%), advice from a physician (8.7%), and concerns about their daughter's health (8.3%) (Table 2). The main reasons for not having a daughter receive the HPV vaccine were concerns about HPV vaccine safety (30.0%), preference to wait until the daughter is older (15.8%), and not enough information to make an informed decision (12.5%). For those parents who indicated that they preferred to have their daughter wait as either their main or one of their reasons (*n* = 337), more than 46.3% said that they felt they needed more safety data, and 27.0% felt that their daughter was not at risk of sexual activity in grade 6 but might be when they were older.

**Table 2:** Reasons for having daughters receive or not receive HPV vaccine.

Reasons for HPV Vaccination Acceptance or Nonacceptance	Main Reason *n* (%)	Any Reason *n* (%)
**Reasons for Having Daughter Receive HPV Vaccine (** ***n*** ** = 1,289)**		
Vaccine is effective in preventing cancer/HPV	619 (48.0)	827 (64.2)
Physician advised me	112 (8.7)	149 (11.6)
Concerned about daughter's health	107 (8.3)	280 (21.7)
Consent to all vaccines, HPV no different	92 (7.1)	158 (12.3)
Public health nurse advised me	80 (6.2)	111 (8.6)
Family member/friend with cervical cancer	40 (3.1)	40 (3.1)
Important to vaccinate prior to sexual activity	32 (2.5)	109 (8.5)
Cervical cancer is a serious disease	30 (2.3)	109 (.8.5)
HPV vaccine is a safe vaccine	23 (1.8)	53 (4.1)
Trust our health care system	18 (1.4)	69 (5.4)
Friend/family/self had cancer	17 (1.3)	17 (1.3)
Benefit outweighed risk	12(0.9)	12 (0.9)
Other	107 (8.3)	
**Main reasons for NOT having daughter receive HPV vaccine (** ***n*** ** = 697)**		
Safety of the vaccine	209 (30.0)	295 (42.3)
Prefer to wait until daughter is older	110 (15.8)	303 (43.5)
Not enough information to make an informed decision	87 (12.5)	148 (21.2)
Vaccine is too new	50 (7.2)	50 (7.2)
Daughter not at risk of cervical cancer	37 (5.3)	88 (12.6)
I do not believe in vaccines, HPV no different	18 (2.6)	25 (3.6)
My physician advised me not to have daughter receive it	17 (2.4)	22 (3.2)
Daughter is too young	14 (2.0)	14 (2.0)
More research needed	13 (1.9)	13 (1.9)
Daughter is not sexually active	13 (1.9)	13 (1.9)
Vaccine is a ploy by pharmaceutical company	12 (1.7)	30 (4.3)
Consent will encourage sexual activity	11 (1.5)	31 (4.4)
Will educate daughter on abstinence and safe sex	10 (1.4)	10 (1.4)
Too many needles	10 (1.4)	21 (3.0)
Other	86 (12.3)	

Internal reliability of the three psychological constructs using Cronbach's alpha were as follows (Table 3): 0.8, overall attitudes to vaccines; 0.7, attitudes of the impact of the HPV vaccine on sexuality; 0.5, seriousness of HPV disease/cervical cancer. In bivariate analysis, age of respondent, country of birth, knowledge of HPV, religious affiliation, history of abnormal Pap smears, and history of cervical cancer were not associated with having a daughter receive the HPV vaccine. Parents with higher levels of education (more than high school diploma/vocational training) were significantly less likely to consent to having their daughter receive the HPV vaccine (63.3% versus 72.9%, *p*<0.01), and parents from non-traditional families (i.e., families not headed by a male and female) were more likely to have their daughters receive the HPV vaccine (71.6% versus 63.1%, *p*<0.01) (Table 4). We did our analysis plan such that variables inputted into the model had to achieve significance in the bivariate model. In multivariate analysis, overall attitudes to vaccines, impact of the HPV vaccine on sexual practices, and childhood vaccine history were predictive of parents having daughter's receive the HPV vaccine in a publicly funded school-based HPV vaccine program. In contrast, having a family with two parents, having three or more children, and having more education was associated with a decreased likelihood of having a daughter receive the HPV vaccine (Table 5).

**Table 3:** Results of psychological construct scales.

Psychological Construct Scale Results	Mean (Standard Deviation)
**Attitudes to vaccines and HPV vaccine overall**	
Childhood vaccines are beneficial	6.1 (1.1)
HPV vaccine is beneficial	5.4 (1.4)
HPV vaccine is effective in preventing cervical cancer	5.3 (1.4)
Immunization is important for public health	6.4 (1.0)
HPV vaccine is a safe vaccine	5.1 (1.5)
Overall mean	5.6 (1.0)
**Influence of HPV vaccine on sexual behaviour**	
Need to give HPV vaccine prior to sexual activity	5.7 (1.6)
HPV vaccine does not lead to earlier sexual activity	5.9 (1.5)
HPV vaccine does not lead to unsafe sexual practices	5.7 (1.6)
HPV vaccine does not lead to more sexual partners	5.9 (1.5)
Safe sex at all times prevents acquisition of HPV	4.7 (1.9)
Overall mean	5.5 (1.1)
**Risk for and seriousness of HPV and cervical cancer**	
Likely for someone you know to get cervical cancer	5.2 (1.7)
Cancer of cervix is a serious illness	6.7 (0.7)
Cervical dysplasia is a serious health concern	6.4 (0.9)
Safe sex at all times prevents acquisition of HPV	6.2 (1.1)
Overall mean	6.1 (0.7)

**Table 4:** Bivariate analysis of uptake rate of HPV vaccine in population.

Characteristics of Respondents	Daughter Received HPV Vaccine *n* (%)
**Respondents' gender**	
Female	1,122 (65.3)
Male	192 (63.8)
**Age of respondents (y)**	
19–29	16 (94.1)
30–39	438 (69.3)
40–49	703 (61.9)
50–59	126 (66.7)
60+	11 (73.3)
**Child received all childhood vaccines**	
Yes (all)	1,280 (67.3)
Yes (some)	29 (35.4)
Unsure	7 (87.5)
No	1 (3.3)
**Ever heard of HPV**	
Yes	1,213 (64.6)
No	105 (71.4)
**History of cervical cancer (self or partner)**	
Yes	61 (76.3)
No	1,231 (64.6)
Unsure/missing	8 (66.6)
**History of abnormal Pap smear (self or partner)**	
Yes	476 (68.0)
No	807 (63.3)
Unsure/missing	16 (69.6)
**Education**	
High school/vocational school	493 (69.1)
Some/complete undergraduate degree/college	700 (62.6)
Postgraduate degree	100 (64.1)
**Family composition**	
Traditional (two parents, male and female)	954 (63.1)
Nontraditional	338 (71.6)
**Number of children**	
One or two children	878 (67.7)
Three or more children	440 (60.4)
**Country of birth**	
Canada	999 (64.7)
England	33 (61.1)
China	10 (66.7)
India	50 (78.1)
Philippines	29 (74.4)
United States	29 (61.7)
Germany	11 (68.8)
Other	157 (63.8)
**Organized religion**	
No religious affiliation	439 (69.5)
Religious affiliation	879 (63.1)

**Table 5:** Multivariate analysis of factors associated with parents' decision to have daughters receive the HPV vaccine in a publicly funded HPV vaccine program.

Factors Associated with HPV Vaccine Uptake	Unadjusted Odds Ratio (95% CI)	Adjusted Odds Ratio (95% CI)
**Childhood vaccine history**		
Received some or no childhood vaccines	1.0	1.0
Received all childhood vaccines	3.9 (2.6–5.9)	1.7 (1.1–2.5)
**Education of respondent**		
High school/vocational school	1.0	1.0
Some/complete undergraduate degree/college	0.7 (0.6–0.9)	0.6 (0.5–0.8)
Postgraduate degree	0.8 (0.6–1.1)	0.6 (0.4–0.9)
**Family composition**		
Nontraditional family composition	1.0	1.0
Traditional family composition	0.7 (0.5–0.8)	0.7 (0.5–0.9)
**Number of children**		
One or two children	1.0	1.0
Three or more children	0.7 (0.6–0.9)	0.8 (0.6–0.9)
**Part of organized religion**		
No religious affiliation	1.0	—
Religious affiliation	0.7 (0.6–0.9)	—
**Attitudes to HPV vaccine and vaccines overall**		
Negative attitudes to vaccines	1.0	1.0
Positive attitudes to vaccines	12.0 (8.8–16.4)	8.5 (6.1–11.9)
**Impact of HPV vaccine on sexual practices**		
Negative impact on sexual practices	1.0	1.0
Limited impact on sexual practices	6.8 (5.3–8.7)	5.1 (3.9–6.7)
**Seriousness of cervical cancer and HPV disease**		
Cervical cancer/HPV disease not serious	1.0	—
Cervical cancer/HPV disease serious	1.7 (1.1–2.6)	—
**Hepatitis B vaccine received with HPV vaccine**		
No hepatitis B vaccine received	1.0	—
Hepatitis B vaccine received	1.1 (1.0–1.2)	—
**Meningitis C vaccine received with HPV vaccine**		
No meningitis C vaccine received	1.0	—
Meningitis C vaccine received	1.0 (1.0–1.1)	—

## Discussion

This program evaluation offers important insights into factors that are associated with parental decisions about receipt of the HPV vaccine in pre-adolescent girls in a program where neither the cost of the vaccine nor access to health care are barriers. In this population-based evaluation of a publicly funded, school-based HPV vaccine program for girls aged 11 y in Canada, parents reported that 65.1% of eligible girls received the first dose of the HPV vaccine, compared to reported receipt of 88.4% for the hepatitis B vaccine, and 86.5% for the meningitis C vaccine. Parents cited vaccine efficacy, advice from a physician, and concerns about daughters' health as the main reasons for choosing to have daughters receive the vaccine. In contrast, concerns about vaccine safety, a desire to wait until their daughter was older, and lack of information were main reasons for not having daughters receive the vaccine. In multivariate modeling, overall attitudes to vaccines and the HPV vaccine, limited concern about the influence of the HPV vaccine on sexual behaviour, and receiving childhood vaccines were associated with having a daughter receive the HPV vaccine. In contrast, family composition (two parents), having more children, and higher education were associated with not having a daughter receive the HPV vaccine. Of note, none of the following factors were associated with decisions to receive the HPV vaccine: religious affiliation, country of birth, or a self-reported history of abnormal Pap smears or cervical cancer.

In a previous study [14], parental intention to have daughters receive the HPV vaccine in British Columbia was 62.8% (95% CI 60.2–65.4), which approximates both the reported parental uptake in this current study at 65.1% and first dose HPV vaccine uptake reported in the provincial clinical immunization record in the province for 2008 of 64.8% [24]. This finding indicates that intention to vaccinate studies can be very useful in planning for actual uptake of the HPV vaccine, albeit with limitations. Comparing the intention to vaccinate [14] with our study, some common factors emerge as key predictors of intention to vaccinate and actual vaccination. These factors included overall attitudes to vaccines and role of the HPV vaccine on sexual behaviour. In our study of actual HPV vaccine uptake, previous actions around vaccines, including childhood vaccine history, were positively associated with the decision to have daughters receive the HPV vaccine. A higher level of parental education and more traditional family composition, including greater numbers of children and two-parent families, were associated with a decision to not have daughters receive the vaccine. These factors were not evident in the intention to vaccinate survey, underscoring the importance of examining actual rather than intended behaviour.

This evaluation has important implications broadly for HPV vaccine policy, because there were neither financial nor organizational barriers to receipt of the HPV vaccine in this program. The vaccine program was fully funded for all girls in grade 6 and was delivered in schools throughout British Columbia as part of a well-established school-based immunization program. Despite this access to the program, almost 35% of parents elected not to have their daughters receive the HPV vaccine. In an examination of parents of almost 3,000 girls aged 12 and 13 y in Manchester, United Kingdom, vaccine uptake was 70.6% for the first dose [20], and parents identified vaccine safety and long term data as a key factor in vaccine refusal. In a qualitative study of 52 parents, Dempsey et al. found that parents identified lack of knowledge, safety, and a perception that their daughter was too young as factors associated with declining of the HPV vaccine [25]. In a study of 153 mothers that included both those intending to have daughters vaccinated and those who had vaccinated their daughters, less education, parental history of a sexually transmitted infection, parental supervision, and acceptance of the vaccine schedule were associated with HPV vaccine acceptance [26]. The findings of these studies echo those found in this study in which parents expressed concerns about the long term safety of the HPV vaccine as a primary reason for refusing to have daughters vaccinated. Parents who did not permit their daughters to receive the vaccine were also concerned about the young age of their daughters, believed the vaccine condoned sexual activity, or believed their daughter was at low risk for acquiring HPV. It is noteworthy that in British Columbia, prior to implementation of the HPV vaccine program, one of the most comprehensive vaccine education programs to date for the province was implemented. These efforts targeted issues such as vaccine safety and efficacy and were delivered in several user-friendly formats including the www.immunizeBC.ca Web site, through DVDs targeted at parents and girls, as well as with pamphlets and brochures and locally held information sessions for parents and providers. In addition, this vaccine was strongly recommended by several independent expert health groups, such as the Canadian National Advisory Committee on Immunizations [27]. However, despite these efforts, many parents still perceived that information was inadequate for them to make an informed decision about HPV vaccination.

In keeping with the findings of two recent studies, this evaluation noted that parents with more education were less likely to consent to their daughters receiving the HPV vaccine [17],[26]. This is a surprising outcome, and in contrast to most studies on vaccine rates in children and maternal education, where higher maternal education is associated with higher childhood vaccine rates [28]. There are several differences to consider as we compare our findings to existing literature. The HPV vaccine program in British Columbia is delivered in optimal conditions with limited barriers, and so several of the issues that may cause lower uptake rates in less-educated parents in other jurisdictions may not be operating for this program. Specifically, the HPV vaccine program in British Columbia is part of a well-established adolescent school-based vaccine program, where vaccines are offered at school, during school hours, by trained health professionals. As a result, parents do not need to get prescriptions, leave work, or arrange to bring children to an office or clinic to receive the vaccine. Parents do not need to pay for the vaccine, so there are no financial constraints for parents. Nurses return to schools several times so that children have the opportunities on other occasions to receive their vaccinations. Our evaluation examined uptake of vaccines in an adolescent as opposed to infant/toddler population, so some of the previous findings and underpinning barriers for infants/toddlers may not be as relevant. This evaluation also examined a newly launched as opposed to a well-established vaccine, and so the factors operating in parental decision making may also be different.

Literature has noted that, in settings with low childhood vaccine uptake rates in less-educated mothers, programmatic structures can reduce the impact of maternal education on vaccine uptake rates. In a recent review by Racine [28], higher maternal education, independent of income and race/ethnicity, was associated with higher child immunization rates. He found, however, that in jurisdictions where there were greater subsidies for childhood vaccines, there was a significantly smaller difference between rates of immunization in children of less versus more educated mothers. This analysis of US data proposed that with increased public funding for vaccines, many of the barriers that create the immunization rate gradient, such as price and availability, decline in their importance, and the advantages offered by maternal education with respect to childhood vaccine receipt are attenuated. In a setting such as British Columbia, where there are even more programmatic advantages such as offering the vaccine in the school setting, the factors that lead to lower uptake rates in less-educated parents in other settings may be diminished by the organization of the adolescent immunization program in the province.

Further research and examination is needed to understand this unique relationship. In a recent qualitative study on Texan parents who opt out of childhood vaccine programs, Gullion et al. noted that the parents were highly educated and reported very sophisticated data collection and information processing from a variety of sources including online sources [29]. Educated parents are often more likely to have access to the Internet and other forms of media compared with less-educated parents in the province, and may feel more comfortable researching the Internet for vaccine information. This research may increase access to some of the Web sites that provide contradictory and potentially inaccurate information about the HPV vaccine and increase parents' concerns about vaccine risks. Highly educated parents may also perceive that they are able to interpret complex scientific and clinical health information and trials independently without the assistance of practitioners. In Gullion's work, parents reported high distrust of the medical community and felt that they were better equipped to conduct research on vaccines and more knowledgeable than the medical practitioners on the topic of vaccines [29]. Educated parents may also have felt more comfortable delaying their daughters' vaccination beyond aged 12 y as they would be able to purchase the vaccine privately in the future, should they choose to do so. Guillon's study noted that parents often felt rushed regarding decisions around vaccines, and so the perceived opportunities for discussion about the attributes and risks of vaccines were limited. Clearly, there is a need for further exploration of this topic to understand why educated parents chose to decline the HPV vaccine for their daughters. As educated parents can often be opinion leaders within their communities and school groups, it is particularly important to consider ways to ensure that these parents have accurate information about this and other vaccines, and appropriately contextualize vaccine risk and safety with the risks and sequelae of the vaccine-preventable disease.

Parents who were concerned about the potential impact of the HPV vaccine on sexual practices were less likely to have their daughters receive the HPV vaccine. Over the past 10 y, British Columbia has had a hepatitis B vaccine program for 11-y-old girls and boys. In the corresponding time period, the Canadian provincial adolescent health survey has reported an improvement in sexual practices in adolescents, with delayed sexual debut, as well as safer sexual practices, despite the availability of a vaccine for a sexually transmitted infection in a publicly funded school program in the province [30]. It will be critical to ensure that parents are aware that provincial data have shown that the use of a vaccine for a sexually transmitted infection does not increase risky sexual behaviour.

The goal of this evaluation was to inform, in real time, vaccine promotion efforts in the province of British Columbia to ensure that educational efforts responded to the concerns of the population. From this survey, it is clear that messaging should continue to focus on the effectiveness of the HPV vaccine, and continue to highlight the established safety of the HPV vaccine, as well as the importance and safety of vaccines in general. Health professionals remain central in influencing parents' decision around the HPV vaccine, and education should also target physicians and nurses to ensure that they also possess accurate information for parents who seek their council. Parents need to be aware that the use of a vaccine for a sexually transmitted infection (hepatitis B) over the past 10 y in British Columbia has not adversely affected the sexual health of adolescents [30]. In contrast, during this same time period, they appear to be making better sexual health decisions.

Limitations of this study include our inability to access parents in two health service areas that account for ∼15% of the population of the province and the use of a telephone methodology. Although there were quality assurance interviews both at training with a random review of telephone calls by supervisors and individual quality assurance reviews for data entry, participants were not surveyed twice. Telephone surveys are biased towards English speakers, and there were 304 potential households who could not participate in this evaluation because of a language barrier. However, this was not a random digit survey, and we were able to use telephone numbers provided to public health services by parents, so biases towards access to land lines should be greatly diminished. Regardless, the reported HPV vaccine uptake rate in this evaluation mirrored the uptake rate reported through the provincial clinical immunization record in the province of 64.8% [24]. With a population-based, randomly selected sample of over 2,000, representing almost 10% of the eligible population for the program, we expect these findings to be highly generalizable and informative for HPV vaccine policies in high-income countries worldwide.

This study is one of the first population-based assessments of factors associated with HPV vaccine uptake in a publicly funded school-based program worldwide. Policy makers need to consider that even with the removal of financial and health care barriers, parents, who are key decision makers in the uptake of this vaccine, still possess some hesitancy to have their daughters receive the HPV vaccine. As populations become less familiar with the diseases that vaccines prevent and the sequelae of these diseases, there is a greater focus on the adverse events associated with vaccines, without the consideration of the morbidity and mortality associated with the disease itself, nor the burden of disease averted by the vaccine [31]. The experience with the HPV vaccine highlights the continued need to ensure that the public is informed and receives credible and clear information about both the scientific evidence for immunizations, as well as information about adverse events associated with vaccines in context. Use of the news media, including the Internet, is essential for connecting with the population, and policy makers must ensure that information speaks broadly to the overall benefits of vaccines at a population and individual level, as well as highlighting the attributes of particular vaccines.

## Abbreviations

CI: confidence interval
HPV: human papillomavirus
iPHIS: integrated Public Health information system
TPB: Theory of Planned Behaviour
